# ACPSEM position paper: dosimetry for magnetic resonance imaging linear accelerators

**DOI:** 10.1007/s13246-023-01223-w

**Published:** 2023-02-20

**Authors:** Jarrad Begg, Urszula Jelen, Zoe Moutrie, Chris Oliver, Lois Holloway, Rhonda Brown, the ACPSEM Magnetic Resonance Imaging Linac Working Group (MRILWG)

**Affiliations:** 1 Department of Medical Physics, Liverpool and Macarthur Cancer Therapy Centre, Liverpool, NSW 2170 Australia; 2 Ingham Institute for Applied Medical Research, Liverpool, NSW 2170 Australia; 3 South Western Sydney Clinical School, University of New South Wales, Liverpool, NSW 2170 Australia; 4 St Vincents Clinic, GenesisCare, Darlinghurst, NSW 2010 Australia; 5 Primary Standards Dosimetry Laboratory, Australian Radiation Protection and Nuclear Safety Agency, Yallambie, VIC 3085 Australia; 6 Centre for Medical Radiation Physics, University of Wollongong, Wollongong, NSW 2522 Australia; 7 Institute of Medical Physics, University of Sydney, Camperdown, NSW 2505 Australia; 8 Australian Clinical Dosimetry Service, Australian Radiation Protection and Nuclear Safety Agency, Yallambie, VIC 3085 Australia

**Keywords:** Reference dosimetry, MRI-linac, MRL, MR-guided radiotherapy, MRgRT, ACPSEM position paper

## Abstract

Consistency and clear guidelines on dosimetry are essential for accurate and precise dosimetry, to ensure the best patient outcomes and to allow direct dose comparison across different centres. Magnetic Resonance Imaging Linac (MRI-linac) systems have recently been introduced to Australasian clinics. This report provides recommendations on reference dosimetry measurements for MRI-linacs on behalf of the Australiasian College of Physical Scientists and Engineers in Medicine (ACPSEM) MRI-linac working group. There are two configurations considered for MRI-linacs, perpendicular and parallel, referring to the relative direction of the magnetic field and radiation beam, with different impacts on dose deposition in a medium. These recommendations focus on ion chambers which are most commonly used in the clinic for reference dosimetry. Water phantoms must be MR safe or conditional and practical limitations on phantom set-up must be considered. Solid phantoms are not advised for reference dosimetry. For reference dosimetry, IAEA TRS-398 recommendations cannot be followed completely due to physical differences between conventional linac and MRI-linac systems. Manufacturers’ advice on reference conditions should be followed. Beam quality specification of *TPR*_20,10_ is recommended. The configuration of the central axis of the ion chamber relative to the magnetic field and radiation beam impacts the chamber response and must be considered carefully. Recommended corrections to delivered dose are ${k}_{{Q}_{msr}{Q}_{0}}^{{f}_{msr}{f}_{ref}}$, a correction for beam quality and ${k}_{\overrightarrow{B},{Q}_{msr}}^{{f}_{msr}}$, for the impact of the magnetic field on dosimeter response in the magnetic field. Literature based values for ${k}_{\overrightarrow{B},{Q}_{msr}}^{{f}_{msr}}$ are given. It is important to note that this is a developing field and these recommendations should be used together with a review of current literature.

## Introduction

The safe clinical introduction and optimal use of MRI-Linacs is a multidisciplinary challenge requiring input from, and collaboration between many professions, hospital departments, and external suppliers. ACPSEM seeks to contribute to meeting this challenge by influencing the quality and delivery of MRI guided radiotherapy (MRgRT). ACPSEM aims to prepare the Medical Physics workforce to do this safely by providing leadership through this position paper.

Radiotherapy outcomes are correlated to the dose accuracy of treatments [1, 2]. Reference dosimetry measurements on a radiotherapy machine should be traceable to a primary standard maintained at a primary standards laboratory to enable the highest accuracy of radiotherapy treatment doses. These methods are well established for linear accelerators where there is no magnetic field. Consistent methods for determining reference dose on new or novel radiotherapy equipment need to be implemented for clinical use.

Magnetic Resonance Imaging Linear Accelerators (MRI-linacs) are radiotherapy devices that combine the imaging system of an MRI scanner and the therapeutic system of a linear accelerator [3–6]. Various systems have been designed with differences in the configuration of the radiation beam and the primary magnetic field (either perpendicular or parallel), as well as different primary magnetic field strengths.

Dose determination on radiotherapy linear accelerators predominately uses Farmer-type chambers calibrated at a primary or secondary standards laboratory. Various factors are used to correct the response of the chamber for environmental influences and any differences between the calibration beam and the clinical beam [7]. Magnetic fields can impact both the dose distribution in media and the measured signal in the Farmer-type chamber, thus specialised methods and corrections for determining the absorbed dose on an MRI-linac are required.

Any mention of particular technologies or models does not imply endorsement by ACPSEM.

## Position paper scope

This position paper will:

- Briefly describe MRI-linacs and the impact of the magnetic field on dose distribution.
- Discuss the impact of the magnetic field on dosimetry equipment including detectors, phantoms and ancillary equipment.
- Describe the reference conditions for dose measurements in perpendicular and parallel MRI-linac systems with magnetic fields up to 1.5 T.
- Focus on ionisation chambers as the predominant dosimeter for reference dosimetry on MRI-linacs. This is due to ionisation chambers being practical, widely used in conventional dosimetry and recommended by the codes of practice for conventional dosimetry. As such, the influence of the magnetic field on ionisation chamber measurements will be discussed.
- Provide a formalism and correction factors for measuring the absorbed dose to water on MRI-linacs.

This recommendation will focus primarily on dosimetry of the commercial systems available for purchase at the time of writing in Australia and New Zealand. For these systems the radiation beam and the primary magnetic field are perpendicular and have magnetic field strengths of either 0.35 or 1.5 T.

Dosimetry in a magnetic field is evolving. The information provided in this document is current at the time of publication. Advancements in dosimetry will likely occur after publication. Literature should be reviewed for any changes in advice.

## MRI-linacs

MRI-linacs can be categorised by the configuration of the radiotherapy beam and primary magnetic field (${B}_{0}$ field). Perpendicular MRI-linacs are configured with the radiation beam perpendicular to ${B}_{0}$. Parallel MRI-linacs are configured with the radiation beam parallel to ${B}_{0}$.

The current perpendicular MRI-linac systems are the MRIdian (Viewray Technologies Inc, Cleveland, OH, USA) [5] and Unity (Elekta Solutions AB, Stockholm, Sweden) [6] systems. The MRIdian system utilises a 0.35 T MRI and a nominal 6 MV Flattening Filter Free (FFF) radiotherapy beam. Further details on the technical design of the MRIdian can be found in Kluter [8]. The Unity system utilises a 1.5 T MRI and a 7 MV FFF radiotherapy beam. Details on the technical design can be found in Raaymakers et al*.* [9].

The current parallel MRI-linac systems are the Aurora RT system from MagnetTX [3] and the Australian MRI-linac [4]. The Aurora RT system utilises a 0.5 T MRI and a 6 MV radiotherapy beam [3]. The Australian MRI-linac uses a bespoke 1 T split bore MRI and a 6 MV linac [10].

The interference between the magnetic field and the dose deposition is dependent on both the magnetic field strength and configuration of the system. Table 1 summarises the properties of each system starting from lowest field strength warranting the least interference. The field size is given in the IEC 61217 co-ordinate system [11].

**Table 1:** Properties of current MRI-linac systems. Field size given in IEC 61217 co-ordinate system [11]. Modified with permission from Jelen and Begg [12]

System (Vendor)	Magnet properties			Configuration	Radiation beam properties		
	Type	Strength	Opening		Nominal energy	SID^+^	Field size (X × Y)/leaf width
MRIdian (Viewray) [5, 8]	Split, super-conducting	0.35 T	70 cm Ø bore	Perpendicular	6 MV FFF	0.9 m	27.4 × 24.1 cm/4.15 mm
Aurora-RT (MagnetTX) [3]	Biplanar, high temp. super-conducting	0.5 T	110 (W) x 60 (H) cm	Parallel (perpendicular possible)	6 MV	1.22 m	30 cm/5 mm
Australian MRI-linac [10]	Open, super-conducting	1.0 T	62 cm Ø bore 50 cm gap	Parallel (perpendicular possible)	4 & 6 MV	1.9–3.3 m	30–50 cm/9.5–16.5 mm
Unity (Elekta) [ 6, 9]	Closed, super-conducting	1.5 T	70 cm Ø bore	Perpendicular	7 MV FFF	1.435 m	57.4 × 22 cm/7.2 mm

^+^SID – source-to-isocentre distance

### Dose deposition in water in a magnetic field

#### Perpendicular configuration

The radiation beam axis, and hence the predominant component of electron motion direction, is perpendicular to the magnetic field. During beam generation this can impact both the electron gun and the trajectory of the electrons in the accelerating waveguide [13–15]. Regions of reduced magnetic field strength, either via magnetic field design or passive shielding, are incorporated into the commercial systems to negate the impact of the magnetic field on beam generation.

**Fig. 1:**
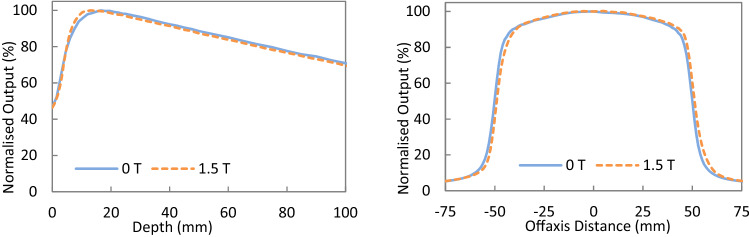
Example PDD (**A**) and crossline profiles (**B**) from the Elekta Unity highlighting differences between 0 and 1.5 T. The PDD and crossline profile show the impact of the 1.5 T magnetic field. Data supplied by J. Arts from Elekta

During dose deposition in the medium, the Lorentz force causes the electron paths between the collisions to become curved, resulting in trajectories with a preferential direction change. Macroscopically, this results in a shifted dose deposition and decreased depth of dose maximum [16]. Figure 1 shows examples of the PDD and profiles measured at 0 and 1.5 T using a PTW Semiflex ionisation chamber (PTW-Freiburg GmbH, Freiburg, Germany) on an Elekta Unity. Details on the use of ion chambers for relative dosimetry measurements can be found in O’Brien et al. [17].

Electrons entering low-density materials (such as lung or air cavity) may curve back towards the interface in a phenomenon termed the electron-return effect (ERE) [18]. ERE from tissue depends on the radiological thickness of the low density region and the electron trajectory radius. Electrons returning to the tissue-lung (air) interface leads to localised dose increases at such interfaces, including at the beam exit surface [18]. The ERE reduces the number of electrons crossing a cavity which results in a lack of electron equilibrium and could result in a reduced dose on the opposite side of a cavity [18].

The Lorentz force sweeps contaminant electrons, generated via interactions in the linac head, away from the incident beam axis in perpendicular MRI-linac systems. This leads to reduction of the skin dose for a beam incident on a perpendicular phantom [19]. Contaminant electrons that have been swept from the incident beam can potentially reach patient surfaces outside the primary beam [20]. Electrons generated via interactions in the patient can also be affected by the Lorentz force. For oblique surface angles, both a decrease and increase in the dose deposited is observed that is dependent on the angle of the surface relative to the primary beam [19, 21]. For surfaces where the Lorentz force causes the electrons to curl toward the surface, an increase in dose is observed. For surfaces where the electrons curl away from the surface, a decrease in dose is observed [21].

In perpendicular MRI-linacs, the magnetic field causes the electron kinetic energy released to be deposited closer to the point of interaction compared to 0 T. This results in a difference in the depth dose deposition curve dependent on the magnetic field strengths. Simulations at multiple magnetic field strengths have been used to investigate the difference in dose deposited per particle history along the central axis. For the 0.35 T field strength of the MRIdian, the dose deposited is reduced by 0.09 ± 0.03% for depths beyond ${d}_{\mathrm{max}}$ [22]. For the 1.5 T field strength of the Unity, the dose deposited is reduced by 0.51 ± 0.03% for depths beyond ${d}_{\mathrm{max}}$ [22, 23]. This difference in dose to water between higher magnetic field strengths and 0 T is part of the correction for the reference dosimetry which will be discussed below.

An important difference between conventional linear accelerators and perpendicular MRI-linacs is the lack of a traditional light field and crosshair for indicating isocentre and dosimetry equipment set-up. Depending on the MRI-linac, either a virtual isocentre outside the MRI bore or EPID images are used to align dosimetry equipment. Details on alignment for the MRIdian and Unity are given in the "Water phantom" section.

#### Parallel configuration

For the parallel MRI-linac configuration the radiation beam axis is parallel to the magnetic field, however, due to scattering, the electrons have motion components orthogonal to the magnetic field. The Lorentz force acts on the vector components of the electron motion that is perpendicular to the magnetic field. This causes the electrons to spiral around the magnetic field direction. Successive energy losses via collisions lead to a shrinking of the helical orbits [24]. Macroscopically, this results in the reduction of the beam penumbra which is especially pronounced in low density materials [25, 26]. Furthermore, the dose deposition perturbations due to density heterogeneities are reduced [24]. Strong parallel magnetic fields reduce the divergence of the contaminate electrons, which are generated in air and in the accelerator, concentrating them around radiation beam axis, which can lead to increased skin doses if not counteracted [27].

For parallel MRI-linacs, such as the Aurora RT or the Australian MRI-linac, the dose deposited at a point is not impacted by the magnetic field provided lateral scatter equilibrium is established [24]. The lack of impact from the magnetic field has been demonstrated by mathematical proofs [24], Monte Carlo simulations [24, 27] and measurements [28]. For regions where there is a lack of electron equilibrium, e.g., lung tissue irradiated by narrow beams, the dose deposition is increased due to the electrons being more predominately forward scatted [29].

It should be noted that the Aurora RT is a commercial system that is not available for purchase in Australia and New Zealand at the time of writing.

## Dosimetry equipment

Reference dosimeters used on MRI-linacs have primarily been ionisation chambers, calorimeters or chemical dosimeters. Additional equipment required includes water phantoms, cables, thermometers and other ancillary equipment. This section discusses the impact of the magnetic field on different equipment. It is also important to understand the safety considerations for different detector equipment in the magnetic field.

### Safety

This section is a brief introduction into MRI safety concepts which should be familiar when using dosimetry equipment in the magnetic environment. A more thorough review on MRI safety is provided in the ACPSEM MRI-linac working group (MRILWG) position paper on MRI-linac safety.

MR safe, MR conditional and MR unsafe [30, 31] are terms/labels given to equipment which may be used in an MRI environment. When purchasing equipment for MRI-linacs it is important that this is considered. The term “MR compatible” can be misleading and wherever possible the modern classifications of equipment safety rating in an MR environment should be used. Physicists should confirm where possible with dosimetry equipment manufacturers the classification of MR safety rating. Several manufacturers provide the MR classification and certification of compliance with international standards such as the American Society for Testing and Materials (ASTM International) [32–35] and the Food and Drug Administration (FDA) [36]. The MR conditional classification for dosimetry equipment generally means the device has been made with only non-ferromagnetic materials. It is also important that ancillary equipment associated with dosimetry (thermometers, barometers, rulers, etc.) are safe for use in the MRI environment. Users should follow the relevant safety protocols for bringing any equipment into the magnetic field. Reference dosimetry equipment should be certified for use in MRI environments where possible.

### Detectors

#### Ionisation chambers

Ionisation chambers measure the charge caused by ionisation events in the air cavity inside the chamber volume. Electrons crossing the cavity experience a change in direction due to the Lorenz force. This change in direction changes the pathlength of the electrons and therefore changes the number of ionisation events occurring within the sensitive volume of the ionisation chamber. Depending on the field strength, the pathlength of the electrons in the cavity of the chamber can either increase, therefore increasing the ionisation events and the charge measured, or decrease, causing a decrease in the charge measured. The effect was first described by Meijsing et al. [37] and has formed the basis for investigations of the dependence of the effect on the relative configuration of the radiation beam, magnetic field and ionisation chambers.

The effective point of measurement (EPoM) of ion chambers in MRI-linacs was observed to shift closer to the central axis and laterally relative to the EPoM for a chamber on a conventional linac at 0 T. The shift in EPoM is due to the Lorentz force giving the secondary electrons a preferential lateral direction [17].

Compounding the ionisation chamber problems, the changed path of the electrons can sometimes cross into the dead volume of the air cavity. The dead volume is the section of the air cavity of the chamber which is defined from the electric field formed between the central and guard electrodes. Ionisations events occurring in the dead volume are not counted in the collected charge. This creates an additional difference relative to 0 T measurements. The impact of the dead volume is dependent on the relative configuration of the chamber, magnetic field and photon beam [38]. Explicit simulation of dead volumes for a chamber has improved Monte Carlo simulation agreement with measurements for corrections applied to chambers for changes in response due to the magnetic field [39].

#### Calorimetry

Calorimetry measures the change in temperature caused by the radiation beam. The two most common types of primary standard calorimeters used in radiotherapy beams measure absorbed dose to graphite or absorbed dose to water. Calorimetry is one of the most accurate methods of determining absorbed dose to water by a primary standards lab. The basic mechanism of calorimetry in radiotherapy is that the energy used to create an ionisation event is transferred into heat resulting in a temperature rise [7]. Calorimeters have been developed and commissioned for use in high and low field perpendicular MRI-linacs [40–42]. The uncertainty of the calorimeter when used at 1.5 T was determined to be the same as under conventional conditions. Comparison between the dose measured for a calorimeter and an ionisation chamber in the magnetic field gives the appropriate absorbed dose to water calibration coefficient for the ionisation chamber in the magnetic field, thus eliminating the need for a correction factor for the magnetic field.

#### Alanine

Alanine is an amino acid that, when irradiated, forms alanine radicals with a concentration relative to the absorbed dose. The alanine powder is pressed into different shaped pellets for use as a dosimeter. Electron Paramagnetic Resonance (EPR) is used to measure the peak-to-peak amplitude of the signal from irradiated pellets. The pellets are near water-equivalent, have a linear response to dose, good reproducibility and small energy dependence [43]. Alanine dosimetry read-out is not immediate requiring a stabilisation period between 24–48 h [44] and specialist EPR equipment. Alanine dosimetry is currently offered as a reference dosimetry audit service for conventional linear accelerators.

Alanine has been investigated as a dose output verification of MRI-linacs via primary standards labs [45, 46] as well as a method for correcting ionisation chambers for the influence of the magnetic field [47]. When used to verify the output calibration, the Alanine pellets are irradiated on the MRI-linac and returned to a primary standards lab for read-out of the dose. This determines the absorbed dose of the MRI-linac [45, 46]. By measuring the output via an ionisation chamber at the same time, the alanine measurement of dose can be used to calibrate the ionisation chambers in the magnetic field at the MRI-linac beam quality [46]. The response of alanine was found to increase with the magnetic field strength by ~ 0.5%/T independently of energy [43]. Air gaps in the alanine holder increased the uncertainty in the measured response at higher magnetic field strengths [46]. Other holders have been designed which do not appear to be influenced by the magnetic field [47].

### Phantoms

#### Water phantom

Water phantoms that meet the requirements of IAEA Code of Practice TRS-398 [7] and are classified as either MR safe or MR conditional are recommended for reference dosimetry. Small 1D phantoms are commonly available for reference dose measurements. Water phantom set up and detector positioning can be challenging without the traditional light field, crosshair and lasers indicating the treatment isocentre.

Water phantoms are designed for placement directly on the treatment couch top with the leading edge of the tank aligned to the couch indexing system at a point outside the bore. Positioning of the detector in the beam relies on the geometric accuracy of couch alignment and travel. MV imaging can also be used for alignment if available. Therefore, other uncertainties in water phantom and detector positioning relative to conventional linacs need to be considered.

Beam characterisation using conventional 3D scanning water tanks generally cannot be performed on MRI-linacs due to size constraints and ferromagnetic components [48]. However, there are now at least two commercially available 3D water scanning tanks specifically designed for use in MRI-linacs. Care must be taken to ensure that the tanks are not subjected to magnetic fields greater than that recommended by the manufacturer and that the chosen dosimeter used in the tank is also suitable for use in a magnetic field.

Water phantom set-up on the two commercially available systems is described below. It is recommended that physicists using this equipment have further vendor training to ensure accurate placement of the water tank.

##### ViewRay MRIdian

The radiation isocentre is indicated by lasers crosshairs at 155 cm along the table outside the bore and is commonly referred to as the virtual isocentre. Initial water phantom set up aligns the phantom and detector with the virtual isocentre. The MRIdian couch can move in all three dimensions to assist with detector alignment and source to surface distance (SSD), within the restrictions set by the 70 cm bore diameter and water phantom height and width. Lasers outside the bore are used to define a virtual isocentre. Once alignment to the virtual isocentre is completed, the couch longitudinal controls send the phantom into the bore to the treatment isocentre [8]. The MRIdian system cannot image the detector. Winston-Lutz tests [49], film placed inside and surrounding a phantom and a multi-axis chamber array [50] have been used to evaluate congruence between the virtual and radiation isocentre. Evaluation of the virtual to radiation isocentre has shown an accuracy of less than 1.0 ± 0.1 mm in three dimensions [49, 50]. Longitudinal studies of the virtual isocentre and radiation isocentre using a daily QA phantom have shown 99.4% of differences were less than 2 mm [51].

##### Elekta Unity

For the Unity MRI-linac, the initial water phantom set up utilises the couch indexing system for longitudinal positioning. Lateral positioning is via spacers placed between the edge of the couch and phantom. The Unity couch is free to move only in the longitudinal direction, thus restricting the options for detector alignment. Judicious choice of lateral placement of the detector within the phantom and phantom positioning on the couch are required for setting up a 1D system in the 70 cm diameter bore. The detector is positioned at the isocentre, which is nominally 14 cm from the couch top (comfort mattress removed) and the water level adjusted to achieve the required SSD. The couch controls send the phantom into the bore to the isocentre at a predetermined longitudinal position. The detector alignment is confirmed using orthogonal MV EPID images. Thus, the stability of the EPID and accuracy of the reference point on the panel is important. Mechanical flex of the EPID on one system was observed to be 0.6 mm in the left–right direction and 0.2 mm in the superior-inferior direction, which was smaller than the flex observed on conventional linacs [52]. MV imaging panel rigidity, once mechanical flex has been corrected for, has been observed to be as small as 0.06 mm [53]. The stability of the location of the reference pixel, and thus the indication of isocentre, is recommended to be within 0.5 mm [54].

#### Solid phantoms

Solid phantoms are not recommended for reference dosimetry in MRI-linacs.

Air gaps surrounding chambers placed in solid water could have an impact on the measured dose of between 0.7%–1.2% for perpendicular aligned MRI-linacs [55]. The difference in measured dose caused by a small air cavity next to the chamber was observed to be dependent on the position of the air bubble relative to the source of radiation and the chamber [56, 57]. The magnitude of the change in the measured charge due to the air gap is dependent on the size of the air gap [57]. The impact of the air gap on the measured charge in a chamber was observed for a range of magnetic field strengths between 0.25 to 2 T [56].

A mitigation strategy suggested to compensate for this is to fill the chamber recess with water [55, 56]. However, adding water around the chamber increases the risk of the chambers getting stuck in the solid phantom due to surface tension, which increases the risk of damage when removing the chamber from the chamber recess.

The effect of air gaps with solid water phantoms on dosimetry has not been investigated for in-line MRI-linacs.

### Ancillary equipment

Ideally the temperature inside the cavity of the chamber should be measured [7]. In practicality, the temperature of the water surrounding the chamber is measured and the chamber is left inside the water tank for enough time that thermal equilibrium is established. Bulb type spirit analogue thermometers are recommended for temperature measurements inside a water tank on an MRI-linac. Digital thermometers should remain outside the bunker. All barometers should remain outside the treatment room.

## Reference dosimetry

This section will focus on the calibration conditions for the commercial systems, the beam quality specification, alignment differences between the photon beam, magnetic field and dosimeter, formalism for calculation of absorbed dose to water in magnetic fields and recommended correction factors for absorbed dose calculations.

### Calibration conditions of the commercial systems

The reference conditions described below have been reported in the literature for the MRIdian and Unity systems. For reference conditions the manufacturers’ advice should be followed. When available, dosimetry protocols should be referred to for reference conditions.

IAEA TRS-398 requires reference conditions of a cylindrical chamber, measurement reference depth of 5 or 10 g/cm^2^ which is dependent on beam energy, reference point of the chamber at the centre of the cavity volume, the reference point of the chamber positioned at the reference depth, a source to surface or source to chamber distance of 100 cm and a field size of 10 cm $\times$ 10 cm. IAEA TRS-398 reference conditions for an isocentic calibration set-up are to place the chamber at the isocentre of the machine. The major difference between the MRI-linacs and a conventional linac are the source to isocentre distances.

Both the Unity and MRIdian systems are also different from IAEA TRS-398 reference conditions in the use of flattening filter free (FFF) beams.

#### ViewRay MRIdian

It is advised to consult the vendor recommendations on calibration set up. MRIdian systems adjust the beam quality, profiles and output of the machine to match the treatment planning system. Reference conditions are modelled in the planning system and the expected machine output determined. Measurements are then acquired on the machine under the references conditions and the output adjusted to match the expected value. The MRIdian is calibrated to deliver 1 cGy/MU with an isocentric setup and a 1.5 cm depth of measurement [58].

The MRIdian has a thin fibreglass connection between either side of the split magnet through which the radiation beam passes [8]. The thin fiberglass panel connecting the split magnet is modelled in the treatment planning system [58].

#### Elekta Unity

It is advised to consult the vendor recommendations on calibration set up. The Unity has been reported to be calibrated under isocentric conditions to deliver:

- 1 cGy/MU at 10 cm depth and SSD = 133.5 cm [59]
- 69.6 cGy per 100 MU, which was the percentage depth dose at 10 cm and resulted in a calibration equivalent to 1 cGy/MU at ${d}_{max}$ at the beam quality of the measured system [60]
- 1 cGy/MU at 5 cm depth and SSD = 138.5 cm [61]

Earlier Unity machines required a fixed MU/min value of 425 MU/min irrespective of calibration conditions. Therefore the calibration point could cause an increase or decrease in the number of pulses from the electron gun to deliver the calibrated dose dependent on depth. Selecting a deeper calibration point would require an increase in the number of electron gun pulses, leading to a potential compromise between faster treatment and stress on linac components. Machine upgrades allowing a wider tolerance on dose rate have resulted in users being able to use deeper calibration points.

The radiation beam from the Unity passes through a helium filled aluminium cryostat and MRI body coil. Whilst these have been designed to be homogeneous, they do attenuate and scatter the beam and are not consistent with IAEA TRS-398 reference conditions. The cryostat and MRI body coil act as a secondary source. Cryostat and MRI body coil transmission has been shown to vary by up to 3% dependent on the gantry angle [, 59, 60] and can be non-uniform due to manufacturing tolerance of the cryostat and gradient coil assembly [60]. A gantry angle cryostat calibration lookup table is included as part of the treatment planning system beam model. The look up table is normalised to the gantry angle of calibration. If a change is made to the calibration gantry angle, then an update to the treatment planning system beam model is recommended. The cryostat calibration lookup table should also be checked after the addition of helium to the system.

It should also be noted that beam calibration for Unity systems was previously performed at a gantry angle of 90° or 270° in order to minimise the effect of varying cryostat liquid helium fill levels on attenuation at gantry 0° [, , 59–61]. When measuring beam output through the side of a water tank, the equivalent thickness of the water tank must be accounted for. The vendor recommendations should be consulted for updated guidelines on the gantry angle used for calibration.

#### Recommended water tank set-up and gantry angles for commercial MRI-linac systems

For both the ViewRay MRIdian and Elekta Unity system, the vendor recommendations should be consulted for updated guidelines on the water tank set-up, ion chamber calibration depth and gantry angle used for reference dosimetry measurements. The MRILWG recommends:

- An isocentric set-up of the reference point of the ion chambers (i.e., central axis of the chamber)
  o For ViewRay MRIdian systems, the reference point for 1 cGy/MU is set by the vendor at 1.5 cm depth and 90 cm source to chamber central axis distance
  p For Elekta Unity systems, the reference point for 1 cGy/MU is recommended to be a minimum of 5 cm. Users should discuss the advantageous and disadvantages of calibrating to a deeper depth with the vendor.
- Ion chamber measurement set-up for validation should be past the point of dose maximum for both systems. A minimum depth of 5 cm should be used as the calibration point.
  o For ViewRay MRIdian systems, the reference conditions will need to be modelled in the treatment planning system to determine an appropriate cGy/MU for comparisons between the TPS and measurements
- The recommend measurement gantry angle is 0°. The practicalities of water tank set-up for irradiation from a gantry angle of 90° or 270° as opposed to a gantry angle of 0° should be considered when selecting a reference gantry angle. For Unity systems, the potential variation in beam attenuation at gantry angles of 0° due to helium fill level in the cryostat should also be considered.
- The recommended field size is as close as physically possible to a 10 $\times$ 10 cm^2^ at isocentre.

### Beam quality specification

The beam quality specification for reference dosimetry in conventional MV photon beams is recommended in IAEA TRS-398 [7] to be based on ${TPR}_{\mathrm{20,10}}$.

To avoid ramp-down of the MRI magnet, the beam quality specifier must be measured in the presence of a magnetic field. Therefore, it should be independent of the magnetic field for reference dosimetry in MRI-linacs. Due to the impact of the static field on secondary electrons and the reduction of electron contamination, significantly higher variation of the of %*dd*(10) as compared to ${TPR}_{\mathrm{20,10}}$ as a function of magnetic field strength has been reported [23, 62]. O’Brien et al*.* [23] also pointed out the practical problem of setting up and measuring *%dd*(10) according to AAPM TG-51 protocol [63] for a machine with a different isocentre distance and a cryostat between the source and measurement point.

Therefore, the existing data suggests that ${TPR}_{\mathrm{20,10}}$ is the preferred beam quality specifier for reference dosimetry in MRI-linac facilities [64].

Although the definition of ${TPR}_{\mathrm{20,10}}$ specifies an SSD of 100 cm, the value of ${TPR}_{\mathrm{20,10}}$ is essentially independent of SDD provided that the field size 10 cm $\times$ 10 cm is maintained [65], which is relevant as both currently commercially available MRI-linac systems have SSDs differing from 100 cm.

Nominal ${TPR}_{\mathrm{20,10}}$ values of 0.648 have been reported for the MRIdian [42] and 0.701 [60] and 0.704 [59] for the Unity have been reported. A range of ${TPR}_{\mathrm{20,10}}$ beam qualities between 0.698 and 0.703 for commissioned Elekta Unity machines have been communicated by the Elekta commissioning team [66].

For Elekta Unity machines, no difference in beam quality due to cryostat fill and attenuation at gantry 90° was observed relative to measurements at gantry 0° [61]. The MRILWG would advise users to check consistency of beam quality between gantry angles as cryostat transmission can vary with gantry angle and cryostat build can be asymmetric [60].

### Configuration of chamber long axis, radiation beam central axis and primary magnetic field

The configuration of the magnetic field and radiation beam results in differences in the dose deposition (see review on dose deposition and the Lorenz force in section above). The dosimeter axis configuration with respect to both the magnetic field and the radiation beam also has an impact on the measured charge collected. Measurements at the effective point of measurement of a Farmer-type chamber, with the long axis of the chamber parallel or perpendicular to the magnetic field direction, show differences of up to 5% in the charge measured by the chamber [48]. This section reviews the different possible configurations of the dosimeter, magnetic field and radiation beam, the differences in correction factors observed and reasons for why different dosimeters exhibit varying dependence on configuration.

**Fig. 2:**
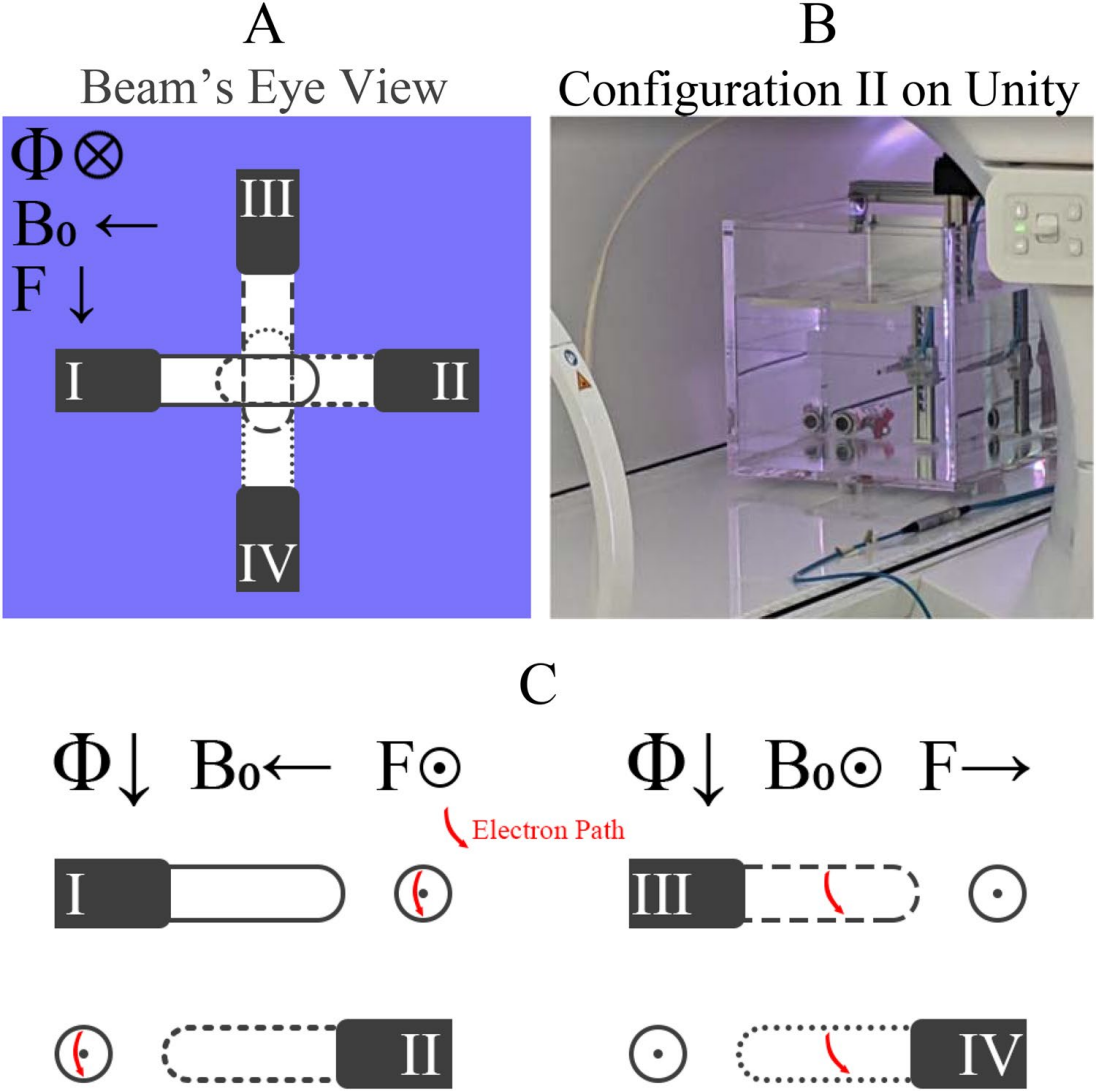
**A** Beam’s eye view of the 4 different configurations in a perpendicular MRI-linac. **B** Chamber aligned in Configuration II on the Unity (Configuration I on the MRIdian). **C** Ionisation chambers as viewed from side on and the impact of the magnetic field on the electrons’ trajectories crossing the chambers

Different configurations have been defined by different authors in the literature. For this review the configuration described below will be used. Figure 2A shows the different configurations described. Figure 2B shows an example of configuration II on a Unity. Figure 2C shows a schematic of the impact of the Lorentz force on the electrons for the different chamber configurations. Configurations I to IV apply to MRI-linacs with the radiation field perpendicular to the magnetic field. Configurations I and II show the cylindrical chamber’s long axis aligned anti parallel and parallel to the magnetic field. This configuration results in the electrons, travelling in the same direction as the photon beam, changing direction around the long axis of the chamber. Configurations III and IV show the long axis of the chamber perpendicular to the magnetic field, again in two opposing directions. These configurations result in electrons, travelling in the same direction as the photon beam, experiencing the Lorenz force and the electrons being directed along the cylindrical chamber axis. For all 4 of these configurations, the change in charged particle pathlength through the chamber results in a change in the charge collected [37].

A major difference between the MRIdian and Unity system is the different directions of the ${B}_{0}$ field [46]. This means that for definitions of configuration I and II to remain consistent, the direction the chamber faces will be different between the systems. For the MRIdian, configuration I results in the chamber tip facing out and configuration II results in the chamber tip facing in. For the Unity, configuration I results in the chamber tip facing into the bore and in configuration II the chamber tip faces out (Fig. 2B).

Differences can occur between configurations III and IV due to differences in the number of electrons directed from the stem into the measurement cavity by the magnetic field (configuration III) relative to electrons directed from water into the measurement cavity (configuration IV) [23].

Configurations I and II have significantly smaller corrections relative to configurations III and IV. Therefore it is recommended to use chamber configuration I or II for reference dosimetry measurements.

### Formalism for determining the absorbed dose to water

The method of determining the dose using an ionisation chamber is defined in different dosimetry standards including the IAEA Technical Report Series 398 [7]. These reports set out formalisms for reference dosimetry and corrections for influence quantities. The dose to water equation for a conventional linac is shown in Eq. 1.

1 $${D}_{w,Q}={M}_{Q}{N}_{{D,w,Q}_{0}}{k}_{{Q,Q}_{0}}$$

where ${D}_{w,Q}$ is the dose to water at a certain beam quality, *Q*, ${M}_{Q}$ is the measured charge at that beam quality under reference conditions and corrected for influence quantities (temperature, pressure, ion recombination and polarity), ${N}_{{D,w,Q}_{0}}$ is the calibration factor in terms of absorbed dose to water in the reference quality *Q*_*0*_ and ${k}_{{Q,Q}_{0}}$ is the correction for the difference in beam quality between the users beam quality, *Q*, and the reference beam quality, *Q*_*0*_. The measurement of influence factors in a magnetic field is discussed below. An ${N}_{{D,w,Q}_{0}}$ for a chamber is determined by sending the chamber to a primary standards lab to be calibrated at the reference beam quality, ${Q}_{0}$. The ${N}_{{D,w,Q}_{0}}$ for a chamber is characterised by measuring the charge collected for a known dose to water under reference conditions, which has been determined via a primary standard such as a calorimeter. Depending on the primary standards lab, the ${N}_{\mathrm{D},\mathrm{w},\mathrm{Q}}$ for a chamber could be determined for several beam qualities. Individual chamber beam quality corrections, ${k}_{{Q,Q}_{0}}$ can be derived from either characterisation of ${N}_{D,w,Q}$ at a primary standard for multiple beam qualities for the chamber, or theoretically derived for the generic chamber type based on the Bragg-Gray cavity theory [7].

At the time of writing the ACPSEM recommends the use of the IAEA TRS-398 [7] for MV dosimetry on linear accelerators. The Australian Radiation Protection and Nuclear Safety Agency (ARPANSA) offer calibration services at both the reference quality Co-60 and multiple MV photon linac energies. Further details on the calibration process and reference dosimetry measurements under non-magnetic conditions can be found in the protocols [7].

As described in the "Calibration Conditions of the Commercial Systems" section above, the commercial systems have different reference beam fields (${f}_{ref}$) from those described in IAEA TRS-398 [7] – primarily differences due to the use of FFF beams. Therefore, modifications to the dose to water equation following IAEA TRS-483 [67] recommendations for machine specific reference fields (${f}_{msr}$) are required.

2 $${D}_{w,{Q}_{msr}}^{{f}_{msr}}={M}_{{Q}_{msr}}^{{f}_{msr}}{N}_{{D,w,Q}_{0}}^{{f}_{ref}}{k}_{{Q}_{msr}{Q}_{0}}^{{f}_{msr}{f}_{ref}}$$

Equation 2 shows the dose to water in the machine specific reference field and beam quality, ${D}_{w,{Q}_{msr}}^{{f}_{msr}}$, is equal to the measured charge at the machine specific reference field at the machine specific beam quality, ${M}_{{Q}_{msr}}^{{f}_{msr}}$, multiplied by the calibration coefficient under standard reference conditions, ${N}_{{D,w,Q}_{0}}^{{f}_{ref}}$, and by the change in response of the ionisation chamber between standard reference conditions and the machine specific reference field, ${k}_{{Q}_{msr}{Q}_{0}}^{{f}_{msr}{f}_{ref}}$. The correction factor ${k}_{{Q}_{msr}{Q}_{0}}^{{f}_{msr}{f}_{ref}}$ incorporates not only the beam quality change but also effects due to volume averaging. Andreo et al*.* [68] provided updated consensus ${k}_{{Q,Q}_{0}}$ data for conventional linacs, for both flattened and FFF beams, based on the end user correcting dosimeter readings by the volume averaging correction, ${k}_{vol}$. Therefore, to apply ${k}_{{Q}_{msr}{Q}_{0}}^{{f}_{msr}{f}_{ref}}$ the ${k}_{{\mathrm{Q},\mathrm{Q}}_{0}}$ is multiplied by the ${k}_{vol}$ as defined in IAEA TRS-483 [67]. Example worksheets for reference dosimetry can be found in IAEA TRS-398 [7].

The magnetic field has an impact on both the dose deposition in water and dosimeter response. Dosimeters can be calibrated directly in the magnetic field or corrections to the standard formalism applied to correct for the influence of the magnetic field.

A direct method of calibrating detectors in MRI-linacs using calorimeters has been reported [40–42, 69]. This method removes any corrections applied to the chamber for beam quality or magnetic fields. However, the method requires specialised calorimetry equipment which is not current available in Australia or New Zealand.

Two different additions to the standard formalisms for correcting for the magnetic field have been proposed. The first correction combines the change in beam quality, between the reference beam quality and the users beam quality (the change caused by the transfer from reference conditions to machine specific fields) and the correction for the changes caused by the magnetic field. The correction has been shown as ${k}_{{Q}_{msr}}^{{B,f}_{msr}}$ [23] or ${k}_{Q}^{mag}$ [62]. The second correction splits the correction into the beam quality correction, ${k}_{{Q}_{msr}{Q}_{0}}^{{f}_{msr}{f}_{ref}}$, and a correction for the impact of the magnetic field on dosimeter response in the magnetic field, ${k}_{\overrightarrow{B},{Q}_{msr}}^{{f}_{msr}}$. Using the second correction method implies that the beam quality correction factors presented in codes of practice are applicable for use in a magnetic field. Monte Carlo simulations of ${k}_{{\mathrm{Q},\mathrm{Q}}_{0}}$ for the Unity beam at 0 T [62] and ion chamber calibrations via calorimetry measurements at ^60^Co and on the Unity at 0 T [69] showed no significant difference in the simulated or measured ${k}_{{\mathrm{Q},\mathrm{Q}}_{0}}$ relative to values presented in the codes of practice. Whilst in general the commercial systems should be tuned close to each other in terms of beam quality, some differences will exist. Using ${k}_{Q}^{mag}$ could add additional uncertainty as the correction uses a predefined beam quality which could be different from the users quality. It is the recommendation of the MRILWG not to use ${k}_{{Q}_{msr}}^{{B,f}_{msr}}$ or ${k}_{Q}^{mag}$ in Australia and New Zealand at this time.

Recommended values for ${k}_{\overrightarrow{B},{Q}_{msr}}^{{f}_{msr}}$ for two types of chambers are given below in Table 3 and 4 for perpendicular MRI-linacs with magnetic field strengths of 0.35 and 1.5 T respectively.

3 $${D}_{B,w,{Q}_{msr}}^{{f}_{msr}}={M}_{\overrightarrow{B},{Q}_{msr}}^{{f}_{msr}}{N}_{{D,w,Q}_{0}}^{{f}_{ref}}{k}_{{Q}_{msr}{Q}_{0}}^{{f}_{msr}{f}_{ref}}{k}_{\overrightarrow{B},{Q}_{msr}}^{{f}_{msr}}$$

Equation 3 adds a correction for the impact of the magnetic field on both the dose deposition and dosimeter response in the magnetic field in the machine specific reference fields, ${k}_{\overrightarrow{B},{Q}_{msr}}^{{f}_{msr}}$ to Eq. 2. The correction for the magnetic field, ${k}_{\overrightarrow{B},{Q}_{msr}}^{{f}_{msr}}$, has a vector component over the B field due to the correction factor having a dependence on the configuration of the photon beam, magnetic field and chamber as described in the "Configuration of chamber long axis, radiation beam central axis and primary magnetic field" section. The correction factor is also dependent on beam quality [62, 70]. ${k}_{\overrightarrow{B},{Q}_{msr}}^{{f}_{msr}}$ corrects the measured charge in the magnetic field, ${M}_{\overrightarrow{B},{Q}_{msr}}^{{f}_{msr}}$, which is set up in the configuration specified by the correction factor. This can then be used to determine the dose to water in the magnetic field for the machine specific reference field and beam quality, ${D}_{B,w,{Q}_{msr}}^{{f}_{msr}}$. The equation given here is identical to the equation presented in de Pooter e*t al.* [64].

The formalism for the magnetic field correction, ${k}_{\overrightarrow{B},{Q}_{msr}}^{{f}_{msr}}$, has been published in a few different notations as summarised in Table 2. Whilst initially the formalisms look different, the corrections for the magnetic field are equivalent (${k}_{\overrightarrow{B},{Q}_{msr}}\equiv {k}_{B}^{{Q}_{msr}}\equiv {k}_{B}\equiv {k}_{\overrightarrow{B},M,Q}\bullet {c}_{\overrightarrow{B}}$).

**Table 2:** Comparison of different notations for magnetic field corrections

Reference	Dose to water	Measured charge	Absorbed dose to water	Beam quality correction	Magnetic field influences	Comments
van Asselen et al*. *[22]	${D}_{w,Q}^{\underset{B}{\to }}$	${M}_{Q}^{\underset{B}{\to }}$	${N}_{{D,w,Q}_{0}}$	${k}_{{Q,Q}_{0}}$	${k}_{\overrightarrow{B},M,Q}\bullet {c}_{\overrightarrow{B}}$	${k}_{\overrightarrow{B},M,Q}=\frac{{M}_{Q}}{{M}_{Q}^{\underset{B}{\to }}}$ effect on IC response, can be measured ${c}_{\overrightarrow{B}}= \frac{{D}_{w,Q}^{\underset{B}{\to }}}{{D}_{w,Q}}$ effect on dose deposition, MC simulated ${{k}_{\overrightarrow{B},Q}=k}_{\overrightarrow{B},M,Q}\bullet {c}_{\overrightarrow{B}}$ Requires MC sim of ${c}_{\overrightarrow{B}}$
Malkov and Rogers [62]	${D}_{W}^{Q}$	$M$	${N}_{D,w}^{{}^{60}Co}$	${k}_{Q}$	${k}_{B}$	${k}_{B}={\left(\frac{{D}_{w}}{{D}_{ch}}\right)}_{Q,0T}^{Q,B}$ MC simulated correction for the difference between 0 T and the magnetic field B, for the dose deposition, ${D}_{w}$, and the dose to the chamber,${D}_{ch}$
O’Brien et al*.* [23]	${D}_{w,{Q}_{msr}}^{{B,f}_{msr}}$	${M}_{{Q}_{msr}}^{B,{f}_{msr}}$	${N}_{{D,w,Q}_{0}}$	${k}_{{Q,Q}_{0}}$	${k}_{{Q}_{msr},Q}^{{f}_{msr}{f}_{ref}}{k}_{B}^{{Q}_{msr}}$	Provided standard reference conditions can be established, then ${Q}_{msr}\equiv Q$ and ${k}_{{Q}_{msr},Q}^{{f}_{msr}{f}_{ref}}=1$ ${k}_{B}^{{Q}_{msr}}= \frac{{D}_{{w,Q}_{msr}}^{B}/{M}_{{Q}_{msr}}^{B}}{{D}_{{w,Q}_{msr}}/{M}_{{Q}_{msr}}}$
de Pooter et al*.* [64]	${D}_{w,Q}$	${M}_{Q}$	${N}_{{D,w,Q}_{0}}$	${k}_{{Q}_{msr},{Q}_{0}}^{{f}_{msr}{f}_{ref}}$	${k}_{\overrightarrow{B},{Q}_{msr}}$	
This paper	${D}_{B,w,{Q}_{msr}}^{{f}_{msr}}$	${M}_{\overrightarrow{B},{Q}_{msr}}^{{f}_{msr}}$	${N}_{{D,w,Q}_{0}}^{{f}_{ref}}$	${k}_{{Q}_{msr},{Q}_{0}}^{{f}_{msr}{f}_{ref}}$	${k}_{\overrightarrow{B},{Q}_{msr}}^{{f}_{msr}}$	

*IC* ionisation chamber, *MC* Monte Carlo

The measured charge is corrected for pressure, recombination, polarity and temperature

### Ion recombination and polarity corrections in the magnetic field

Polarity and ion recombination are influence quantities that affect the ionisation chamber response and need to be corrected for when measuring the absorbed dose to water [7]. The influence of the magnetic field on polarity and recombination correction has been investigated for perpendicular MRI-linacs at both high [48, 69] and low [42] magnetic field strengths. Polarity and recombination corrections have been found to be independent of the magnetic field strength [48, 69].

### ***Values for ***${{\varvec{k}}}_{\overrightarrow{{\varvec{B}}},{{\varvec{Q}}}_{{\varvec{m}}{\varvec{s}}{\varvec{r}}}}^{{{\varvec{f}}}_{{\varvec{m}}{\varvec{s}}{\varvec{r}}}}$*** factors***

Most of the early publications reporting on ${k}_{\overrightarrow{B},{Q}_{msr}}^{{f}_{msr}}$ factors have some flaws caused by the lack of knowledge about peculiarities of dosimetry in magnetic fields which have been realized at a later stage or present insufficient details. A review of existing data, employing stringent selection criteria, has been conducted by de Pooter et al. [64]*.* for the most common ionisation chambers, at the two field strengths corresponding to those of the commercially available MRI-linacs, and for different chamber, magnetic field and beam configurations (shown in Fig. 2).

Table 3 and 4 show the best available ${k}_{\overrightarrow{B},{Q}_{msr}}^{{f}_{msr}}$ data for ionisation chambers in perpendicular MRI-linacs as presented by de Pooter e*t al.* [64]. There are only two chamber types included as this is a relatively new area of research and there is a very limited amount of data available. The correction factors presented are for the beam qualities of the MRIdian and Unity respectively. A Monte Carlo study showed a change in ${k}_{\overrightarrow{B},{Q}_{msr}}^{{f}_{msr}}$ of less than 0.5% between beam qualities of ${TPR}_{20/10}$ values of 0.6790 (6 MV Linac) and 0.7028 (7 MV MRI-linac) [70]. From this it can be concluded that the change in ${k}_{\overrightarrow{B},{Q}_{msr}}^{{f}_{msr}}$ is small within the beam quality variability of the systems. Only ${k}_{\overrightarrow{B},{Q}_{msr}}^{{f}_{msr}}$ data for configuration I and II are reproduced here. If the ionisation chamber is placed in a perpendicular configuration (III and IV in Fig. 2) then the deviation from unity of the ${k}_{\overrightarrow{B},{Q}_{msr}}^{{f}_{msr}}$ factor significantly increases. The average magnitude of ${k}_{\overrightarrow{B},{Q}_{msr}}^{{f}_{msr}}$ presented in de Pooter et al. [64]*.* for IBA FC65-G (IBA Dosimetry GmbH, Schwarzenbruck, Germany) and PTW 30013 chambers in perpendicular MRI-linacs with B = 0.35 T is 0.9744 and ranges from 0.9649 to 0.9830. For the case of perpendicular MRI-linacs with B = 1.5 T, the average ${k}_{\overrightarrow{B},{Q}_{msr}}^{{f}_{msr}}$ presented in the perpendicular configuration for the two chamber types is 0.9594 and ranges from 0.9512 to 0.9700 [64].

**Table 3:** ${k}_{\overrightarrow{B},{Q}_{msr}}^{{f}_{msr}}$ data for perpendicular linacs with B = 0.35 T and ${TPR}_{\mathrm{20,10}}$ = 0.648 for the PTW 30013 chamber. All values are determined in the parallel configurations (see Fig. 2). The data is reproduced from de Pooter et al. [64]

Chamber	${k}_{\overrightarrow{B},{Q}_{msr}}^{{f}_{msr}}$	$u({k}_{\overrightarrow{B},{Q}_{msr}}^{{f}_{msr}})$) %	Method of determination	Configuration (see Fig. 1)	Reference
PTW30013	0.9970	0.3000	Monte Carlo	II	Spindeldreier et al*.* [38]
	0.9965	0.3000	Monte Carlo	I	Spindeldreier et al*.* [38]
	0.9957	0.1000	Monte Carlo	I	Malkov and Rogers [62]

**Table 4:** ${k}_{\overrightarrow{B},{Q}_{{\varvec{m}}{\varvec{s}}{\varvec{r}}}}^{{f}_{{\varvec{m}}{\varvec{s}}{\varvec{r}}}}$ data for perpendicular linacs with B = 1.5 T and an average ${TPR}_{\mathrm{20,10}}$ = 0.701 for the PTW 30013 and IBA FC65-G chambers. All values are determined in the parallel configurations (see Fig. 2). The data is reproduced from de Pooter et al. [64]

Chamber	${k}_{\overrightarrow{B},{Q}_{msr}}^{{f}_{msr}}$	u$u({k}_{\overrightarrow{B},{Q}_{msr}}^{{f}_{msr}})$) %	Method of determination	Configuration (see Fig. 1)	Reference
PTW30013	0.9930	0.3000	Monte Carlo	I	Spindeldreier et al*.*[38]
	0.9920	0.3000	Monte Carlo	II	Spindeldreier et al*.* [38]
	0.9963	0.1600	Monte Carlo	I	Pojtinger et al
	0.9881	0.1000	Monte Carlo	II	Malkov and Rogers [62]
	0.9920	0.2000	Ramp up/down	I	van Asselen et al*.* [22]
	0.9850	0.3400	Calorimetry Cross Cal	I	de Prez et al*.* [69]
	0.9980	1.0000	Alanine	I & II	Billas and Duane [71]
FC-65G	0.9970	0.3000	Ramp up/down	I	van Asselen et al*.* [22]
	0.9917	0.1000	Monte Carlo	II	Malkov and Rogers [62]
	0.9950	0.3400	Calorimetry Cross Cal	I	de Prez et al*.* [69]
	1.003	1.0000	Alanine	I	Billas and Duane [43]

Due to the smaller perturbation in the response of the ionisation chambers in magnetic fields in the parallel configuration, this configuration is recommended for reference dosimetry. The question then becomes which ${k}_{\overrightarrow{B},{Q}_{msr}}^{{f}_{msr}}$ value to use from the published range. All the values do not agree within their uncertainties so this would suggest that some of the uncertainties in these ${k}_{\overrightarrow{B},{Q}_{msr}}^{{f}_{msr}}$ values are underestimated and thus not fully understood.

Table 5 presents the average ${k}_{\overrightarrow{B},{Q}_{msr}}^{{f}_{msr}}$ values for orthogonal linacs with B = 1.5 and 0.35 T for PTW 30,013 and IBA FC65-G chambers. Taking a value of ${k}_{\overrightarrow{B},{Q}_{msr}}^{{f}_{msr}}$ from Table 5 could be viewed as an appropriate choice for users of the two currently available commercial MRI-linacs. The estimated uncertainty in these values is relatively large as the number of measurements and calculations which contribute to these results is quite small. The uncertainty in these values will decrease in the future as more ${k}_{\overrightarrow{B},{Q}_{msr}}^{{f}_{msr}}$ will be determined and better understanding of the dead volumes is incorporated into Monte Carlo simulations to calculate ${k}_{\overrightarrow{B},{Q}_{msr}}^{{f}_{msr}}$. As time progresses it is up to the user to monitor the literature for progress in the measurement and simulation of ${k}_{\overrightarrow{B},{Q}_{msr}}^{{f}_{msr}}$ values for reference ionisation chambers to ensure that the most appropriate value for their situation is applied when performing reference dosimetry.

**Table 5:** Average ${k}_{\overrightarrow{B},{Q}_{{\varvec{m}}{\varvec{s}}{\varvec{r}}}}^{{f}_{{\varvec{m}}{\varvec{s}}{\varvec{r}}}}$ for perpendicular linacs with B = 1.5 and 0.35 T for PTW 30013 and IBA FC65-G chambers. All values are for the parallel configuration (see Fig. 2). The data is an average of the values presented in de Pooter et al. [64]

Chamber	B	${k}_{\overrightarrow{B},{Q}_{msr}}^{{f}_{msr}}$	u($u({k}_{\overrightarrow{B},{Q}_{msr}}^{{f}_{msr}})$) %
PTW30013	0.35 T	0.996	0.200
PTW30013	1.5 T	0.992	0.400
IBA FC65-G	1.5 T	0.997	0.400

## Future research

Reference dosimetry on MRI-linacs is an emerging field. Devices used to measure dose in the magnetic field based on temperature changes are limited to a few sites worldwide [40–42]. Further development of these devices could improve the accuracy and reproducibility of the measured signal or improve portability so that the devices can be transferred between MRI-linacs enabling direct measurement of dose and chamber calibration in the magnetic field.

The current protocols have been developed with a focus on correcting ion chambers for a response change caused by the magnetic field. As has been shown by the understanding of dead volumes in chambers [38, 39], increased knowledge of chambers characteristics can be fed back into improving Monte Carlo simulations. Improvements in chamber simulations could also have an impact on simulations in 0 T fields.

The ion chambers used in the current protocols are those that are recommended in the conventional linac protocols (e.g., IAEA TRS-398 [7]). Research into alternative dosimeters might find a more suitable dosimeter, that fits the requirements of a reference grade dosimeter as per protocols and that does not require correction for magnetic fields.

Further investigations into correction factors are required as shown by the limited data available on alternative chambers and differences between physically measured and simulated correction factors presented in Table 3 and 4. Alternative dosimeters to standard reference ion chambers such as smaller volume chambers [72], microDiamond and microSilicon type detectors [73] have also been evaluated. Similar to conventional linacs, the output factors for small fields and the response of detectors under non-equilibrium conditions is an emerging area of research [74].

The protocol above was developed considering current MRI-linacs. Development of new MRI-linacs or indeed MRI-Protons [75] might require this protocol to be revisited.

## Summary of recommendations

The following are a summary of recommendations for perpendicular MRI-linacs. The recommendations are current as of the time of writing, however all users are advised to consult current literature and MRI-linac specific reference dosimetry protocols that are developed post publication of this position paper.

- Ion chambers are to be used for reference dosimetry on MRI-linacs provided appropriate correction factors are available and applied
- Ion chambers are calibrated at Primary Standards Laboratories under standard conditions
- TRS-398 methodologies are to be followed for correction of influence quantities (polarity, recombination, temperature and pressure)
- Bulb type spirit thermometers are recommended for temperature measurements inside a water tank on an MRI-linac
- Barometers can be left outside the room for pressure measurements
- Water tanks are to be used for all reference dosimetry measurements. Solid water phantoms are not recommended for dosimetry measurements
- Beam Quality is measured using ${TPR}_{\mathrm{20,10}}$ with the chamber positioned at the machine isocentre
- If a change is made to the calibration gantry angle on the Elekta Unity machines, then an update of the cryostat lookup table and beam model is recommended
- The reference point of an ion chamber (i.e., central axis) is recommended to be set-up isocentrically
- The measurement point should at minimum 5 cm depth in water. Details on the calibration conditions for both the ViewRay MRIdian and Elekta Unity are given in the "Recommended water tank set-up and gantry angles for commercial MRI-linac systems" section
- The reference beam gantry angle is recommended to be 0°
- A field size as close to a 10 $\times$ 10 cm^2^ field should be used
- Configuration I or II (Chamber long axis parallel to primary magnetic field and perpendicular to the radiation central axis – see the "Configuration of chamber long axis, radiation beam central axis and primary magnetic field" section) is recommended for reference dosimetry set-up on perpendicular MRI-linacs
- An addition to the standard reference dosimetry equation, ${k}_{\overrightarrow{B},{Q}_{msr}}^{{f}_{msr}}$, is used to account for the impact of the magnetic field on the measured response of an ion chamber
- Values for ${k}_{\overrightarrow{B},{Q}_{msr}}^{{f}_{msr}}$ are detailed in "[Sec Sec28]"

## References

[1] Brahme A (1988). Accuracy requirements and quality assurance of external beam therapy with photons and electrons. Acta Oncol.

[2] Mijnheer BJ, Battermann JJ, Wambersie A (1987). What degree of accuracy is required and can be achieved in photon and neutron therapy?. Radiother Oncol.

[3] Fallone BG (2014). The rotating biplanar linac-magnetic resonance imaging system. Semin Radiat Oncol.

[4] Keall PJ, Barton M, Crozier S, Australian MRI-Linac Program including contributors from Ingham Institute for Applied Medical Research, Illawarra Cancer Care Centre, Liverpool Hospital, Stanford University, Universities of Newcastle, Queensland, Sydney, Western Sydney, and Wollongong (2014). The Australian magnetic resonance imaging-linac program. Semin Radiat Oncol.

[5] Mutic S, Dempsey JF (2014). The ViewRay system: magnetic resonance-guided and controlled radiotherapy. Semin Radiat Oncol.

[6] Lagendijk JJ, Raaymakers BW, van Vulpen M (2014). The magnetic resonance imaging-linac system. Semin Radiat Oncol.

[7] INTERNATIONAL ATOMIC ENERGY AGENCY (2000) Absorbed Dose Determination in External Beam Radiotherapy, Technical Reports Series No. 398, IAEA, Vienna https://www.iaea.org/publications/5954/absorbed-dose-determination-in-external-beam-radiotherapy

[8] Klüter S (2019). Technical design and concept of a 0.35 T MR-Linac. Clin Transl Radiat Oncol.

[9] Raaymakers BW, Jurgenliemk-Schulz IM, Bol GH, Glitzner M, Kotte A, van Asselen B, de Boer JCJ, Bluemink JJ, Hackett SL, Moerland MA, Woodings SJ, Wolthaus JWH, van Zijp HM, Philippens MEP, Tijssen R, Kok JGM, de Groot-van Breugel EN, Kiekebosch I, Meijers LTC, Nomden CN, Sikkes GG, Doornaert PAH, Eppinga WSC, Kasperts N, Kerkmeijer LGW, Tersteeg JHA, Brown KJ, Pais B, Woodhead P, Lagendijk JJW (2017). First patients treated with a 1.5 T MRI-Linac: clinical proof of concept of a high-precision, high-field MRI guided radiotherapy treatment. Phys Med Biol.

[10] Liney GP, Dong B, Weber E, Rai R, Destruel A, Garcia-Alvarez R, Manton DJ, Jelen U, Zhang K, Barton M, Keall P, Crozier S (2018). Imaging performance of a dedicated radiation transparent RF coil on a 1.0 Tesla inline MRI-linac. Physics in Medicine & Biology.

[11] International Electrotechnical Commission IEC 1217:1996 Radiotherapy Equipment - Cooridinates, movments and scales

[12] Jelen U, Begg J (2019). Dosimetry needs for MRI-linacs. J Phys: Conf Ser.

[13] Constantin DE, Fahrig R, Keall PJ (2011). A study of the effect of in-line and perpendicular magnetic fields on beam characteristics of electron guns in medical linear accelerators. Med Phys.

[14] St Aubin J, Steciw S, Fallone BG (2010). Effect of transverse magnetic fields on a simulated in-line 6 MV linac. Phys Med Biol.

[15] Whelan B, Holloway L, Constantin D, Oborn B, Bazalova-Carter M, Fahrig R, Keall P (2016). Performance of a clinical gridded electron gun in magnetic fields: Implications for MRI-linac therapy. Med Phys.

[16] Raaymakers BW, Raaijmakers AJ, Kotte AN, Jette D, Lagendijk JJ (2004). Integrating a MRI scanner with a 6 MV radiotherapy accelerator: dose deposition in a transverse magnetic field. Phys Med Biol.

[17] O'Brien DJ, Dolan J, Pencea S, Schupp N, Sawakuchi GO (2018). Relative dosimetry with an MR-linac: Response of ion chambers, diamond, and diode detectors for off-axis, depth dose, and output factor measurements. Med Phys.

[18] Raaijmakers AJ, Raaymakers BW, Lagendijk JJ (2005). Integrating a MRI scanner with a 6 MV radiotherapy accelerator: dose increase at tissue-air interfaces in a lateral magnetic field due to returning electrons. Phys Med Biol.

[19] Keyvanloo A, Burke B, Warkentin B, Tadic T, Rathee S, Kirkby C, Santos DM, Fallone BG (2012). Skin dose in longitudinal and transverse linac-MRIs using Monte Carlo and realistic 3D MRI field models. Med Phys.

[20] Hackett SL, van Asselen B, Wolthaus JWH, Bluemink JJ, Ishakoglu K, Kok J, Lagendijk JJW, Raaymakers BW (2018). Spiraling contaminant electrons increase doses to surfaces outside the photon beam of an MRI-linac with a perpendicular magnetic field. Phys Med Biol..

[21] Raaijmakers AJ, Raaymakers BW, van der Meer S, Lagendijk JJ (2007). Integrating a MRI scanner with a 6 MV radiotherapy accelerator: impact of the surface orientation on the entrance and exit dose due to the transverse magnetic field. Phys Med Biol.

[22] van Asselen B, Woodings SJ, Hackett SL, van Soest TL, Kok JGM, Raaymakers BW, Wolthaus JWH (2018). A formalism for reference dosimetry in photon beams in the presence of a magnetic field. Phys Med Biol.

[23] O'Brien DJ, Roberts DA, Ibbott GS, Sawakuchi GO (2016). Reference dosimetry in magnetic fields: formalism and ionization chamber correction factors. Med Phys.

[24] Bielajew AF (1993). The effect of strong longitudinal magnetic fields on dose deposition from electron and photon beams. Med Phys.

[25] Alnaghy SJ, Begg J, Causer T, Alharthi T, Glaubes L, Dong B, George A, Holloway L, Metcalfe P (2018) Technical Note: Penumbral width trimming in solid lung dose profiles for 0.9 and 1.5 T MRI-Linac prototypes. Med Phys 45 (1):479–487. doi:10.1002/mp.12680 10.1002/mp.12680 29156098

[26] Oborn BM, Ge Y, Hardcastle N, Metcalfe PE, Keall PJ (2016). Dose enhancement in radiotherapy of small lung tumors using inline magnetic fields: A Monte Carlo based planning study. Med Phys.

[27] Oborn BM, Metcalfe PE, Butson MJ, Rosenfeld AB, Keall PJ (2012). Electron contamination modeling and skin dose in 6 MV longitudinal field MRIgRT: Impact of the MRI and MRI fringe field. Med Phys.

[28] Begg J, Alnaghy SJ, Causer T, Alharthi T, George A, Glaubes L, Dong B, Goozee G, Keall P, Jelen U, Liney G, Holloway L (2019). Technical Note: Experimental characterization of the dose deposition in parallel MRI-linacs at various magnetic field strengths. Med Phys.

[29] Oborn BM, Gargett MA, Causer TJ, Alnaghy SJ, Hardcastle N, Metcalfe PE, Keall PJ (2017). Experimental verification of dose enhancement effects in a lung phantom from inline magnetic fields. Radiother Oncol.

[30] Kanal E, Barkovich AJ, Bell C, Borgstede JP, Bradley WG, Froelich JW, Gimbel JR, Gosbee JW, Kuhni-Kaminski E, Larson PA, Lester JW, Nyenhuis J, Schaefer DJ, Sebek EA, Weinreb J, Wilkoff BL, Woods TO, Lucey L, Hernandez D (2013). ACR guidance document on MR safe practices: 2013. J Magn Reson Imaging.

[31] Greenberg TD, Hoff MN, Gilk TB, Jackson EF, Kanal E, McKinney AM, Och JG, Pedrosa I, Rampulla TL, Reeder SB, Rogg JM, Shellock FG, Watson RE, Weinreb JC, Hernandez D, Safety: ACoM (2020). ACR guidance document on MR safe practices: updates and critical information 2019. J Magn Reson Imaging.

[32] ASTM International ASTM F2052–15: Standard Test Method for Measurement of Magnetically Induced Displacement Force on Medical Devices in the Magnetic Resonance Environment. 10.1520/F2052-15

[33] ASTM International ASTM F2503–20: Standard Practice for Marking Medical Devices and Other Items for Safety in the Magnetic Resonance Environment. 10.1520/F2503-20

[34] ASTM International ASTM F2213–17: Standard Test Method for Measurement of Magnetically Induced Torque on Medical Devices in the Magnetic Resonance Environment. 10.1520/F2213-17

[35] ASTM International ASTM F2119–07(2013), Standard Test Method for Evaluation of MR Image Artifacts from Passive Implants. 10.1520/F2119-07R13

[36] Food and Drug Administration Testing and Labeling Medical Devices for Safety in the Magnetic Resonance (MR) Environment. https://www.fda.gov/regulatory-information/search-fda-guidance-documents/testing-and-labeling-medical-devices-safety-magnetic-resonance-mr-environment

[37] Meijsing I, Raaymakers BW, Raaijmakers AJ, Kok JG, Hogeweg L, Liu B, Lagendijk JJ (2009). Dosimetry for the MRI accelerator: the impact of a magnetic field on the response of a Farmer NE2571 ionization chamber. Phys Med Biol.

[38] Spindeldreier CK, Schrenk O, Bakenecker A, Kawrakow I, Burigo L, Karger CP, Greilich S, Pfaffenberger A (2017). Radiation dosimetry in magnetic fields with Farmer-type ionization chambers: determination of magnetic field correction factors for different magnetic field strengths and field orientations. Phys Med Biol.

[39] Pojtinger S, Kapsch RP, Dohm OS, Thorwarth D (2019). A finite element method for the determination of the relative response of ionization chambers in MR-linacs: simulation and experimental validation up to 1.5 T. Phys Med Biol.

[40] de Prez L, de Pooter J, Jansen B, Woodings S, Wolthaus J, van Asselen B, van Soest T, Kok J, Raaymakers B (2019). Commissioning of a water calorimeter as a primary standard for absorbed dose to water in magnetic fields. Phys Med Biol.

[41] D'Souza M, Nusrat H, Iakovenko V, Keller B, Sahgal A, Renaud J, Sarfehnia A (2020). Water calorimetry in MR-linac: direct measurement of absorbed dose and determination of chamber kQmag. Med Phys.

[42] Krauss A, Spindeldreier CK, Klüter S (2020). Direct determination of for cylindrical ionization chambers in a 6 MV 0.35 T MR-linac. Phys in Med & Biol.

[43] Billas I, Bouchard H, Oelfke U, Shipley D, Gouldstone C, Duane S (2020). Alanine dosimetry in strong magnetic fields: use as a transfer standard in MRI-guided radiotherapy. Phys Med Biol.

[44] McEwen M, Sharpe P, Vörös S (2015). Evaluation of alanine as a reference dosimeter for therapy level dose comparisons in megavoltage electron beams. Metrologia.

[45] McEwen M, Malkov V, Muir B, Sarfehnia A (2020). Verification of the Output Calibration of MR-Linac Beams Using Reference Alanine Dosimeters. MEDICAL PHYSICS.

[46] Billas I, Bouchard H, Oelfke U, Duane S (2021). Traceable reference dosimetry in MRI guided radiotherapy using alanine: calibration and magnetic field correction factors of ionisation chambers. Phys Med & Biol.

[47] Pojtinger S, Nachbar M, Ghandour S, Pisaturo O, Pachoud M, Kapsch RP, Thorwarth D (2020). Experimental determination of magnetic field correction factors for ionization chambers in parallel and perpendicular orientations. Phys Med Biol.

[48] Smit K, van Asselen B, Kok JG, Aalbers AH, Lagendijk JJ, Raaymakers BW (2013). Towards reference dosimetry for the MR-linac: magnetic field correction of the ionization chamber reading. Phys Med Biol.

[49] Wen N, Kim J, Doemer A, Glide-Hurst C, Chetty IJ, Liu C, Laugeman E, Xhaferllari I, Kumarasiri A, Victoria J, Bellon M, Kalkanis S, Siddiqui MS, Movsas B (2018). Evaluation of a magnetic resonance guided linear accelerator for stereotactic radiosurgery treatment. Radiother Oncol.

[50] Latifi K, Moros EG, Zhang G, Harrison L, Feygelman V (2019). A method to determine the coincidence of MRI-guided linac radiation and magnetic isocenters. Technol Cancer Res Treat.

[51] Mittauer KE, Dunkerley DAP, Yadav P, Bayouth JE (2019). Characterization and longitudinal assessment of daily quality assurance for an MR-guided radiotherapy (MRgRT) linac. J Appl Clin Med Phys.

[52] Torres-Xirau I, Olaciregui-Ruiz I, Baldvinsson G, Mijnheer BJ, van der Heide UA, Mans A (2018). Characterization of the a-Si EPID in the unity MR-linac for dosimetric applications. Phys Med & Biol.

[53] Woodings SJ, de Vries JHW, Kok JMG, Hackett SL, van Asselen B, Bluemink JJ, van Zijp HM, van Soest TL, Roberts DA, Lagendijk JJW, Raaymakers BW, Wolthaus JWH (2021). Acceptance procedure for the linear accelerator component of the 1.5 T MRI-linac. J Appl Clin Med Phys.

[54] Roberts DA, Sandin C, Vesanen PT, Lee H, Hanson IM, Nill S, Perik T, Lim SB, Vedam S, Yang J, Woodings SW, Wolthaus JWH, Keller B, Budgell G, Chen X, Li XA (2021). Machine QA for the Elekta Unity system: a report from the Elekta MR-linac consortium. Med Phys.

[55] Hackett SL, van Asselen B, Wolthaus JW, Kok JG, Woodings SJ, Lagendijk JJ, Raaymakers BW (2016). Consequences of air around an ionization chamber: are existing solid phantoms suitable for reference dosimetry on an MR-linac?. Med Phys.

[56] Agnew J, O'Grady F, Young R, Duane S, Budgell GJ (2017). Quantification of static magnetic field effects on radiotherapy ionization chambers. Phys Med Biol.

[57] O'Brien DJ, Sawakuchi GO (2017). Monte Carlo study of the chamber-phantom air gap effect in a magnetic field. Med Phys.

[58] Attieh C (2022) Personal Communication on the ViewRay MRIdian dosimetry and TPS calculations

[59] Snyder JE, St-Aubin J, Yaddanapudi S, Boczkowski A, Dunkerley DAP, Graves SA, Hyer DE (2020). Commissioning of a 1.5T Elekta Unity MR-linac: a single institution experience. J Appl Clin Med Phys.

[60] Woodings SJ, Bluemink JJ, de Vries JHW, Niatsetski Y, van Veelen B, Schillings J, Kok JGM, Wolthaus JWH, Hackett SL, van Asselen B, van Zijp HM, Pencea S, Roberts DA, Lagendijk JJW, Raaymakers BW (2018). Beam characterisation of the 1.5 T MRI-linac. Phys Med Biol.

[61] Powers M, Baines J, Crane R, Fisher C, Gibson S, Marsh L, Oar B, Shoobridge A, Simpson-Page E, Van der Walt M, de Vine G (2022). Commissioning measurements on an Elekta Unity MR-Linac. Phys Eng Sci Med.

[62] Malkov VN, Rogers DWO (2018). Monte Carlo study of ionization chamber magnetic field correction factors as a function of angle and beam quality. Med Phys.

[63] Almond PR, Biggs PJ, Coursey BM, Hanson WF, Huq MS, Nath R, Rogers DW (1999). AAPM's TG-51 protocol for clinical reference dosimetry of high-energy photon and electron beams. Med Phys.

[64] de Pooter JA, Billas I, de Prez LA, Duane S, Kapsch RP, Karger C, van Asselen B, Wolthaus JWH (2020). Reference dosimetry in MRI-linacs: evaluation of available protocols and data to establish a code of practice. Phys Med Biol.

[65] Budgell G, Gohil P, Agnew J, Berresford J, Billas I, Duane S (2019). Absolute Calibration of the Elekta Unity MR Linac Using the UK Code of Practice for High-Energy Photon Dosimetry. World Congress on Medical Physics and Biomedical Engineering 2018.

[66] Arts J (2022) Personal Communication on beam quality values on commissioned Elekta Unity MRLs.

[67] Palmans H, Andreo P, Huq MS, Seuntjens J, Christaki KE, Meghzifene A (2018). Dosimetry of small static fields used in external photon beam radiotherapy: summary of TRS-483, the IAEA–AAPM international code of practice for reference and relative dose determination. Med Phys.

[68] Andreo P, Burns DT, Kapsch RP, McEwen M, Vatnitsky S, Andersen CE, Ballester F, Borbinha J, Delaunay F, Francescon P, Hanlon MD, Mirzakhanian L, Muir B, Ojala J, Oliver CP, Pimpinella M, Pinto M, de Prez LA, Seuntjens J, Sommier L, Teles P, Tikkanen J, Vijande J, Zink K (2020). Determination of consensus k Q values for megavoltage photon beams for the update of IAEA TRS-398. Phys Med & Biol.

[69] de Prez L, Woodings S, de Pooter J, van Asselen B, Wolthaus J, Jansen B, Raaymakers B (2019). Direct measurement of ion chamber correction factors, k Q and k B, in a 7 MV MRI-linac. Phys Med Biol.

[70] Pojtinger S, Nachbar M, Kapsch RP, Thorwarth D (2020). Influence of beam quality on reference dosimetry correction factors in magnetic resonance guided radiation therapy. Phys Imaging Radiat Oncol.

[71] Billas I, Duane S (2018) Report on dose measurements on the Elekta Unity MR-linac at Christie Hospital performed by NPL. NPL Report IR 50

[72] Delfs B, Blum I, Tekin T, Schönfeld A-B, Kranzer R, Poppinga D, Giesen U, Langner F, Kapsch R-P, Poppe B, Looe HK (2021). The role of the construction and sensitive volume of compact ionization chambers on the magnetic field-dependent dose response. Med Phys.

[73] Tekin T, Blum I, Delfs B, Schönfeld A-B, Kapsch R-P, Poppe B, Looe HK (2020). The dose response of high-resolution diode-type detectors and the role of their structural components in strong magnetic field. Med Phys.

[74] Blum I, Tekin T, Delfs B, Schönfeld A-B, Kapsch R-P, Poppe B, Looe HK (2021). The dose response of PTW microDiamond and microSilicon in transverse magnetic field under small field conditions. Phys Med & Biol.

[75] Oborn BM, Dowdell S, Metcalfe PE, Crozier S, Mohan R, Keall PJ (2017). Future of medical physics: real-time MRI-guided proton therapy. Med Phys.

